# Evaluation of Physical and Mental Health in Adults Who Underwent Limb-Lengthening Procedures with Circular External Fixators During Childhood or Adolescence †

**DOI:** 10.3390/children11111322

**Published:** 2024-10-30

**Authors:** Alessandro Depaoli, Marina Magnani, Agnese Casamenti, Marco Ramella, Grazia Chiara Menozzi, Giovanni Gallone, Marianna Viotto, Gino Rocca, Giovanni Trisolino

**Affiliations:** Unit of Pediatric Orthopedics and Traumatology, IRCCS Istituto Ortopedico Rizzoli, 40136 Bologna, Italy; alessandro.depaoli@ior.it (A.D.);

**Keywords:** limb lengthening, Ilizarov, external fixation, congenital, pediatric, patient-reported outcomes, Short Form 36, Stanmore Limb Reconstruction Score

## Abstract

Background: Lower limb length discrepancy (LLD) in children and adolescents, often due to congenital or acquired conditions, is treated to achieve limb equality and alignment, optimizing function and minimizing cosmetic concerns for an active adulthood. This study evaluated the Health-Related Quality of Life (HRQoL) and physical functioning of adults who underwent unilateral limb lengthening with circular external fixators (EFs) in childhood. Methods: Fifty patients treated at a median age of 14.9 years completed the Short Form 36 (SF-36) and Stanmore Limb Reconstruction Score (SLRS) questionnaires in adulthood, with a median follow-up of 8.9 years. Results: Among the 50 patients, 38 underwent a single limb lengthening (21 tibia, 12 femur, 5 both), while 12 required multiple cycles. The median residual LLD was 0.4 cm, with 12 patients (24%) having over 2 cm. Complications occurred in 67% of procedures, mainly due to prolonged healing. Physical and mental health scores were significantly lower than normative data. The mean Physical Component Summary was 52.2 ± 7.2 (*p* = 0.20). The mean Mental Component Summary was 43.9 ± 8.6 (*p* = 0.001), notably lower in congenital LLD cases. Many SLRS items (Pain, Social, Physical Function, Work, and Emotions) strongly correlated with SF-36 items. Conclusions: Adults treated with distraction osteogenesis for congenital LLD show normal physical but lower mental health scores compared to peers. Lengthening procedure characteristics did not significantly impact mental health. Routine psychological and social assessments are recommended to prevent long-term distress by providing appropriate support.

## 1. Introduction

Lower limb length discrepancy (LLD) and associated deformities are common concerns during childhood and adolescence, resulting from congenital conditions or acquired factors like sepsis sequelae, trauma involving growth plates, tumors, or rare genetic disorders [1]. The overarching goal in treating children with LLD is to enable an active adult lifestyle with optimal function, minimal musculoskeletal pain, and minimal cosmetic concerns [2]. Decisions about LLD treatment in children are typically made collaboratively by parents and healthcare professionals during childhood, encompassing both operative and non-operative approaches [3]. Traditionally, orthopedic surgeons strive for nearly normal alignment, equal limb length, and a typical gait pattern through surgical interventions. This objective commonly entails preserving the foot and salvaging the limb through lengthening procedures, a preference typically shared by both patients and parents when compared to the alternative of amputation and fitting a prosthesis [4,5]. However, limb salvage may entail repeated interventions, prolonged treatments, and a high risk of complications or failure, with the added challenge of an occasionally uncertain final functional outcome. These challenges can profoundly impact the child or adolescent [3].

Given the complexity and duration of limb lengthening procedures, which often require multiple stages of intervention and rehabilitation, it is essential to evaluate not only the clinical outcomes but also the patient’s overall well-being and life satisfaction. Patient-Reported Outcome Measures (PROMs) provide a structured way to capture the patient’s perspective on their physical functioning, mental health, and social participation throughout and after the treatment [6]. In the context of lower limb lengthening, PROMs are especially valuable in assessing the long-term impacts of treatment on the patient’s quality of life, including how they perceive the success of the procedure and how it affects their daily activities, social interactions, and emotional state [7]. They facilitate the quantification of improvement achieved or desired from a therapeutic intervention, allowing comparisons of diverse therapeutic approaches and evaluation against general or target population standards. Investigating PROMs streamlines informed decision-making among therapeutic strategies and reveals aspects of health and well-being most affected by a pathological condition—some (such as loss of self-esteem, feelings of inadequacy, anxiety or depression, and social impact) challenging to clinically detect but crucial to address in treating a pathology. In the case of lower limb lengthening procedures, where outcomes are not limited to physical measurements like leg length but extend to how patients feel about their appearance, functionality, and emotional health, PROMs help bridge the gap between clinical success and real-life patient satisfaction.

Limited information currently exists on the long-term quality of life for patients who underwent limb-lengthening procedures due to congenital or acquired deformities in childhood or adolescence. Several studies highlighted the psychosocial distress in individuals with skeletal deformities or LLD due to self-perception of physical appearance [8,9]. However, the achieved correction may not always enhance the patient’s perceived well-being as expected [10]. Residual disfiguring scars, joint stiffness, residual LLD or deformities, along with syndromic associations highlighting the disparity between expected and actual outcomes, partially contribute to this psychological distress. Coping with rare and disfiguring skeletal conditions during childhood and adolescence, along with a prolonged and challenging clinical journey involving repeated hospitalizations, multiple surgeries, and the need to undergo intense procedures like circular external fixation, can weigh heavily and leave lasting psychological distress into adulthood. This study investigates Health-Related Quality of Life (HRQoL) and physical functioning in adults who underwent unilateral limb lengthening with circular external fixation (EF) during childhood, comparing their outcomes with age- and gender-matched normative data from the general population.

## 2. Materials and Methods

This was a retrospective study with prospectively collected data that included pediatric and adolescent patients who underwent one or more lower limb lengthening procedures using circular EF at a single institution between January 2009 and December 2021 (NCT06519175). The surgical technique used in this study has been comprehensively detailed in a prior publication [11]. Patients with LLD who underwent unilateral lengthening of the lower limb with circular EF before the age of 18 were invited to complete two specific questionnaires to evaluate their quality of life upon reaching legal adulthood (>18 years). Exclusion criteria comprised: age > 18 years at the time of the lengthening procedure, and age < 18 when responding to the questionnaires, bilateral lengthening procedures (e.g., achondroplasia), or conditions other than LLD (e.g., acute fractures), patients treated with intramedullary lengthening nail, patients with insufficient radiographic data (at least one preoperative full-length standing radiograph of the lower limbs in the anteroposterior projection and one postoperative radiograph of the segment after EF removal were considered sufficient), and patients with incomplete or partially answered questionnaires. The following patient data were extracted from medical records: sex, affected side, family history, underlying pathologies, comorbidities, and LLD etiology. Any previous surgeries performed outside our institution were documented (e.g., hemiepiphysiodesis, corrective osteotomies, etc.). Specifics of the surgical lengthening procedures were assessed, and the Total Treatment Time (TTT) and the Healing Index (HI) were calculated for each intervention [11]. All data were recorded blindly by two independent authors (A.D. and A.C.). Complications were assessed according to Lascombes’ classification [12], with HI > 45 days/cm defined as a major complication (grade IIIa) but considered separately from the other complications [11]. The requirement for orthoses, or assistive devices was also recorded. The presence of painful symptoms or functional limitations, along with the assessment of quality of life, was assessed with the following Patient-Reported Outcome Measures (PROMs): the Italian version of the Short Form 36 (SF-36) and the Stanmore Limb Reconstruction Score (SLRS) [7,13,14,15]. While the SF-36 is a generic, widely used, multidimensional tool divided into 8 scales, designed to assess overall health status and capture the impact of a disease on various dimensions of quality of life, the SLRS was recently designed specifically for patients undergoing limb reconstruction surgery. Telephonic and email communication was established with all patients by a single author (A.C.), who proposed the questionnaires. Call details were recorded and divided into three primary groups: patients who did not respond to the phone call or email, patients who declined to recount their experience or voice concerns, and patients who expressed willingness to complete the questionnaires. Those who consented to participate in the questionnaires received a form containing the SF-36 and the SLRS. A section for additional comments was included at the end of each form.

Statistical analyses were performed using STATA (version 17.0) based on the data collected in Excel 2021 (Microsoft Corporation, San Jose, CA, USA). The normality of the distribution of continuous variables was tested using the Shapiro–Wilk test and comparisons were made with Mann–Whitney U tests or Student’s *t* tests depending on the data distribution. Results were expressed as mean (±standard deviation—SD) for continuous variables with normal distribution, median with first and third quartile (Q1–Q3) and/or complete range for non-normally distributed variables, and as numbers with associated percentages for categorical variables. Univariable and multivariable analysis with linear and logistic regression were performed to assess the influence of baseline variables (e.g., age at surgery, sex, preoperative LLD, etiology, and age at survey) and surgical variables (e.g., bone healing, complications, and residual LLD) on the outcomes (SF-36 and SLRS). For SF-36, the Physical Component Summary (PCS) and Mental Component Summary (MCS) were calculated according to the method developed by Ware et al. [14,16]. The most recent normative data by age and sex available for a European country were used for comparison, using a *t*-test for comparison of means and grouping the patients by number of SD from normative data (above −1, between −1 and −2 and below −2) [17]. The relationship strength among variables was assessed using the correlation coefficient (absolute adjusted R-squared value): R-squared < 0.3: None or very weak effect size; 0.3 ≤ R-squared < 0.5: Weak or low effect size; 0.5 ≤ R-squared < 0.7: Moderate effect size; and R-squared ≥ 0.7: Strong effect size [18]. A difference was considered statistically significant for a *p*-value less than 0.05.

## 3. Results

### 3.1. Patients Included and Demographics

**Figure 1:**
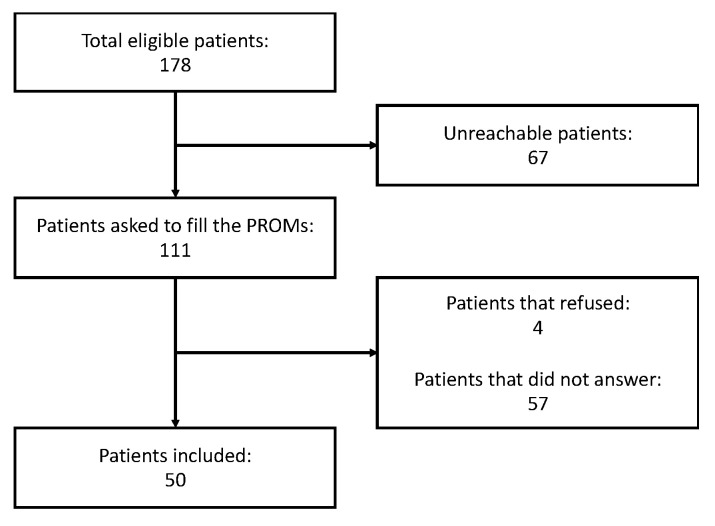
Flowchart of inclusion of patients.

Based on clinical records, a cohort of 178 eligible patients was initially identified for inclusion in this study. Unfortunately, 67 of these patients were unreachable due to various issues, such as missing contact information, non-functioning numbers, or unresponsive calls. Of the 111 patients successfully contacted by telephone and invited to participate by completing questionnaires, 4 declined to participate, and 50 patients (28% of the entire series) completed the questionnaires and were included in the final analysis (see flowchart in Figure 1). The remaining 57 patients, who initially agreed to participate, ultimately did not return their completed questionnaires, despite the follow-up reminders. No statistically significant differences were observed in primary outcomes between these groups (see details in Table S1 in Supplementary Materials).

In the responders’ cohort, 38 patients underwent a single limb lengthening procedure. Among them, 21 patients received lengthening in the tibial segment, 12 patients in the femoral segment, and 5 patients in both segments simultaneously. Additionally, 12 patients required two or more lengthening cycles, and in 6 of these cases, a single limb segment was treated multiple times. Overall, 70 segments were treated, 29 femurs and 41 tibiae, at a median patient age of 14.9 years (range 7.0–17.3, see details in Table 1). The median preoperative LLD at the first lengthening procedure was 6.0 cm (range 3–20 cm), corresponding to a median 3.6% of height (range 2–13%).

### 3.2. Surgical Parameters and Outcomes

Most lengthening procedures (93%) were performed with the traditional Ilizarov circular frame, while five procedures (7%) were performed with hexapod EFs. Median follow-up was 8.9 years since the last EF removal (range 2.0–13.5 years). Overall, the median HI was 51 days/cm (range 24–151 days/cm), and the median TTT was 247 days (range 135–604 days). There were no statistically significant differences observed in both the HI and the TTT when comparing congenital and acquired etiologies (*p*-value > 0.42). Likewise, no significant differences were found between the femur and tibia for both HI and TTT (*p*-value > 0.36).

Residual LLD had a median value of 0.4 cm (range 0.0–9.0 cm). A total of 38 patients (76%) had a residual LLD up to 2 cm, while 12 patients (24%) had a residual LLD exceeding 2 cm. Preoperative LLD showed a correlation with residual LLD after all lengthening procedures (Spearman’s rho = 0.60, *p*-value = 0.001). In particular, patients with preoperative LLD of more than 4.5% of height had a significantly higher prevalence of a residual LLD of more than 2 cm, rising from 7% to 41% (*p*-value = 0.006).

### 3.3. Complications

Twenty-three lengthening procedures (33%) had no complications, while, among the remaining forty-seven procedures, seventy-one complications were observed, which were all major ones except for sixteen cases of minor complications. HI was higher than 45 days/cm in thirty-eight procedures (54%), and among nineteen of them (27% of total procedures), high HI was the only complication observed. Details about complications are reported in Table 2.

Revision surgery was required during or after eight lengthening procedures. One case of pin infection required surgical debridement and revision of local pins. One case of fracture of femoral regenerate after EF removal was treated with cast immobilization and healed with severe procurvatum deformity, which then required corrective osteotomy. One case of early consolidation required revision of the osteotomy site to complete lengthening. A patient, after simultaneous lengthening of the femur and tibia, developed severe stiffness of the knee and ankle, which were treated with femoral and tibial osteotomies and contralateral epiphysiodesis. A case of non-union was treated with open fixation with a plate and an autograft from iliac crest eight months after EF removal. In one case of femoral lengthening, EF was removed during the lengthening phase for an infection and the patient was then treated with an opening-wedge osteotomy to treat the residual shortening and valgus deformity. In one case of femoral lengthening, residual valgus deformity required corrective osteotomy.

### 3.4. PROMs

**Figure 2:**
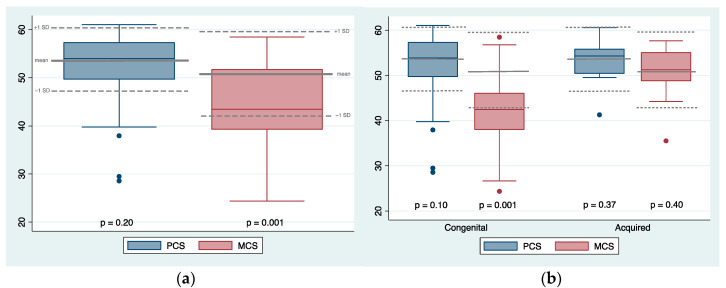
Distribution of Physical Component Summary (PCS, in blue) and Mental Component Summary (MCS, in red) results compared with normative data by age and sex, in which the thick gray line represents the mean score and the dashed gray lines the ±1 SD values. Results of MCS were significantly lower (**a**), especially among patients treated for congenital etiologies of LLD (**b**).

The median age at survey was 23.1 (range 18.5–29.3). Among the SF-36 items, physical function (PF), general health (GH), social function (SF), role-emotional (RE) and mental health (MH) were significantly lower than normative data (*p*-value = 0.001). Conversely, results in role-physical (RP), bodily pain (BP), and vitality (VT) were comparable with normative data (*p*-value > 0.130). The mean Physical Component Summary (PCS) was 52.2 ± 7.2 (range 28.6–61.1), with no significant difference from normative data for age (*p*-value = 0.20). The Mental Component Summary (MCS) had a mean value of 43.9 ± 8.6 (range 24.4–58.5), significantly lower than normative data for age (*p*-value = 0.001, see Figure 2a). Sixteen percent of patients had a PCS score more than 1 standard deviation (SD) below the normative data, and 6% had a score more than 2 SD below. For the MCS, 42% of patients scored more than 1 SD below the normative data, while 10% scored more than 2 SD below (see details in Figure S1 in Supplementary Materials). This difference was even more evident in patients affected by congenital LLD, in which 51% had MCS results below −1 SD, and 12% below −2 SD (see Figure 2b). Detailed results for each SF-36 item are reported in Table 3.

Preoperative LLD showed a very weak impact on BP and PCS (adjusted R-squared 0.07 with *p* = 0.038), while residual LLD showed a weak influence on PF items and on PCS (adjusted R-squared 0.11 with *p* = 0.025). Age at questionnaire showed a weak influence on the GH item (adjusted R-squared 0.07 with *p* = 0.034). None of the pre- and postoperative variables considered showed influence on the MCS results (see Table 4 for details).

The results of the SLRS for the entire cohort are reported in Table 5. Many of the SLRS items (Pain, Social, Physical Function, Work, and Emotions) showed a strong correlation with one or more items combined with the SF36 (adjusted R-squared > 0.72, *p* = 0.001, see details in Table S3 in Supplementary Material). Hygiene in the SLRS showed a medium correlation with SF-36 Physical Function (adjusted R-squared = 0.53, *p* = 0.001). Sleep, Leisure, Future, and Cosmetic showed no more than weak correlations with all SF36 items, including the PCS and MCS (adjusted R-squared < 0.30). Conversely, PF, BP, SF, and RE showed strong correlations with other items of the SLRS (adjusted R-squared > 0.77, *p* = 0.001), while RP, GH, VT, and MH showed medium correlations with other items of the SLRS (adjusted R-squared 0.41–0.55, *p* = 0.001). The PCS showed a strong correlation with SLRS Physical Function and Work (adjusted R-squared 0.63, *p* = 0.001), while the MCS showed a strong correlation with SLRS Social and Emotions (adjusted R-squared 0.73, *p* = 0.001).

## 4. Discussion

Our study explores the HRQoL and physical functioning of young adults who underwent unilateral correction and lengthening procedures using a circular external fixator for various reasons during childhood. In our series, 82% reached normal values in the PCS, while only 46% did so in the MCS. The main finding that emerges is the high prevalence of residual psychological distress despite achieving clinical-functional milestones, a result that was found almost only among patients affected by congenital LLD. Patients with acquired LLD had both PCS and MCS scores comparable to the healthy population, similar to data on the treatment of post-traumatic LLD reported by Schep et al., who found normal values of SF-36 items in fifteen adults who underwent distraction osteogenesis at a mean follow-up of 9 years [19]. On the other hand, most patients affected by congenital etiologies of LLD had MCS scores below −1 SD from normative data. Their MCS items were frequently at least 8.9 below the mean value for age and sex. For comparison, the effect of depression is a reduction in MCS between 9.3 and 12.7 [14].

This high prevalence of psychological distress among patients treated for congenital LLD has never been reported in the literature (see Table S4 in Supplementary Materials). It apparently contrasts with the findings of Moraal et al., who reported no effect of etiology on perceived competence at an average follow-up of seven years [20]. Some authors stated that congenital LLD often involves greater limb discrepancies and requires longer treatment protocols [21]. Based on the available data, congenital and acquired LLD had comparable treatment protocols and complication rates, so we cannot confirm this hypothesis. The low MCS, since it does not correlate with outcome variables of the lengthening procedure, could be influenced by many different factors, such as the psychological impact of the underlying condition, the family’s coping strategies, the variability in patients’ personalities, and/or the psychological support provided before and during the treatment phase.

The absence of dedicated psychological support throughout the lengthening procedure could account for the unpredictable variability in long-term MCS outcomes observed in our case series. In 1990, Hrutkay and Eilert advocated for professional preoperative psychological support for all adolescents undergoing the lengthening procedure using the Ilizarov technique [22]. Since then, a gradually increasing body of evidence has emerged, underscoring the importance of this type of support. Ghoneem et al. observed overall normal psychological scores in 45 patients treated between 3 and 18 years of age who were given a thorough psychological preparation during the lengthening procedure [23]. Similarly, Ramaker et al. found no psychological issues caused by the Ilizarov lengthening procedure if patients and parents were given preoperative assessment for depression and anxiety and support during the months of the procedure [24]. As a consequence, lower scores in the MCS may have been the consequence of insufficient support for children and adolescents to cope with their condition. Niemelä et al. observed a higher prevalence of behavior problems in patients with LLD of various etiologies compared to healthy children of the same age [25]. They also clearly demonstrated that strong support in coping with the condition can significantly enhance the Ilizarov lengthening procedure’s effectiveness in improving children’s behavior [25]. In summary, growing patients with LLD, especially those with congenital causes, are often vulnerable both psychologically and socially. As such, treatment should focus not only on correcting the length discrepancy but also on helping the child build confidence and a positive body image. Martin et al. recommended putting a lot of effort into helping the patients during the first month of lengthening, in which they found the highest level of distress for the patients [26].

Some studies have suggested that the age at which patients undergo their first lengthening procedure may impact their ability to cope with the process. Ghoneem et al. found that 83% of patients treated between the ages of 6 and 12 were willing to undergo the Ilizarov method again, compared to only 45% of those treated between 13 and 18 years old. Similarly, Niemelä et al., who repeatedly monitored psychological distress before, during, and up to one year after external fixator removal, observed higher levels of anxiety and depression in children treated between 10 and 12 years of age. In our analysis, however, we found no significant influence of age at the first procedure on the MCS score [23].

Preoperative and residual LLD had a weak effect on PCS, and based on the available data, did not significantly impact the MCS [6,8,10,24,25,26,27]. Our findings partially contrast with the study by Moraal et al., where seven years after Ilizarov limb lengthening, patients showed normal psychosocial functioning, self-esteem, and perceived competence, exhibiting quality of life scores similar to norm groups, with exceptions of reduced gross motor function, lower vitality, and increased pain [20]. Notably, a residual LLD greater than 2 cm remained a significant factor in long-term follow-up, leading to a reported decline in quality of life. Other studies have confirmed this impact, emphasizing that both the initial limb length inequality—whether congenital or acquired—and contributing factors such as obesity can significantly affect overall quality of life, including mental and social aspects [10]. Ramaker et al. observed in a cohort of 26 patients that 87% of them would undergo the lengthening procedure again, but the authors could not identify a precise complication and/or residual issue associated with bad experiences in the remaining 13% [24]. One patient from our case series wrote: “I hope the doctors who put their hands on me suffer as much as I did”. An important takeaway from this study is that any young patient undergoing a lengthening procedure may express a similar dissatisfaction, but it is impossible to predict who will feel this way. Even with a flawless technique, minimal time in the frame, and no complications, this dissatisfaction may still arise. The difference among families and/or other type of social support may explain this unpredictability.

The SF-36 was used in this study due to its widespread use in assessing quality of life, including in patients undergoing limb reconstruction with circular EF [28]. Its results can also be compared with normative data from large, up-to-date global cohort studies [29]. With its incorporation of eight domains, it offers a comprehensive assessment of the patient’s overall well-being. Moreover, the method of evaluating outcomes derived from SF36 questionnaires through comparison with normative data from the general population has become an established approach in various analogous studies. This method proves particularly valuable when dealing with scenarios where a pre-operative SF36 score is unavailable. This comparative analysis enhances the interpretability of the SF36 outcomes, providing a contextually meaningful understanding of the impact of interventions in the absence of pre-operative baseline measurements. Kaastad et al. compared the HRQoL, assessed using SF-36, among limb-deficient individuals with normative data from the general health population. They observed diminished physical functioning, heightened bodily pain, and decreased general health and emotional role. These outcomes align with our study, suggesting an enduringly low quality of life into adulthood for children who underwent limb lengthening [29]. Other multidimensional questionnaires have been used to evaluate patients with lower limb discrepancies requiring lengthening with external fixation, including the EuroQol, PedsQL, PODCI, and WHOQoL-BREF [10,29,30,31].

The main criticism of these questionnaires is that they are not specific to any particular condition or treatment, which may result in some aspects of well-being, emotional impact, and the psychosocial effects of circular external fixation going underexplored. Recent systematic reviews found that existing PROMs for patients undergoing limb reconstruction with circular external fixation do not fully capture the specific health outcomes relevant to this group, underscoring the need for tailored PROMs [9,32]. There is a clear need to develop new PROMs specifically for patients with congenital limb length discrepancies who require lengthening with external fixation. The SLRS was developed for this purpose, and we tested it against the SF-36, finding a strong correlation between the two questionnaires. However, the SLRS, recently introduced in a pilot study, has only undergone face validity testing. Its reliability, responsiveness, precision, and criterion validity have yet to be tested, and it has not been applied to large populations undergoing limb reconstruction surgery [7].

More recently, the LIMB-Q and the LIMB-Q Kids questionnaires have been validated in multiple languages and show promise as the most specific tools for assessing LLD [33,34,35,36]. Also, the PROLLIT (Patient-Reported Outcome Measure for Lower Limb Reconstruction) study group developed a conceptual framework outlining six key domains important to patients undergoing limb lengthening with circular external fixation: pain, self-perception, work and finances, daily lifestyle and functioning, emotional well-being, and support. Some of these domains, particularly “support”, are poorly represented in current PROMs, prompting the development of new, condition-specific tools [37]. However, further research is needed to gather more data for accurate interpretation. Monitoring changes in quality of life throughout the entire lifespan, from childhood to adulthood, is undoubtedly challenging, particularly for these patients. The decision to undergo such a stressful treatment is often made by caregivers, adding another layer of complexity to the process.

### Limitations

Several limitations should be noted. The retrospective design of the study introduces the potential for recall bias, which may have influenced the results. Additionally, the comparison with a preoperative assessment and/or a control group of healthy adults of the same age is only partially addressed by using normative data adjusted for age and sex. However, the SF-36 is not validated for children under 15 years of age, making it impossible to compare results for more than 50% of the patient cohort. Furthermore, the heterogeneity of LLD etiologies with varying levels of severity may serve as a confounding factor. However, many of these conditions are exceedingly rare, even in a large pediatric orthopedic center, making it challenging to assemble a consistent case series. Another significant source of bias was the low patient adherence to PROMs, with only 28% of the initial case series (and just 45% of reachable patients) returning completed forms. This limitation is challenging to address, as low adherence to completing PROMs (Patient-Reported Outcome Measures) via phone or email has been reported, with response rates falling below 30% in some studies [38]. This issue is especially pronounced in cases involving complex and painful treatments, where patients may be inclined to mentally distance themselves from the experience [38].

## 5. Conclusions

Adults who underwent distraction osteogenesis for congenital limb length discrepancy (LLD) during infancy or adolescence show normal physical health scores, but lower mental health scores compared to age- and sex-matched normative data. The specific characteristics of the lengthening procedures did not significantly influence mental health outcomes. Consequently, we recommend the routine assessment of psychological and social factors before, during, and after the lengthening process using age-appropriate, validated questionnaires, preferably in a prospective study setting. This approach aims to identify and prevent long-term psychological distress by providing appropriate coping support.

## Figures and Tables

**Table 1:** Patients’ descriptives and surgical outcomes by etiology. In the table body, bold text is used to specify overall results for congenital causes, acquired causes and total, italic text is used to specify results for subgroups in proximal limb hypoplasia, distal limb hypoplasia and skeletal dysplasia. N = number; LPs = lengthening procedures; Q1–Q3 = first and third quartile values; LLD = lower limb length discrepancy; HI = healing index; TTT = total time of treatment; CFD = congenital femoral deficiency; DDH = developmental dysplasia of the hip; CPMBT = congenital posteromedial tibial bowing; NF1 = neurofibromatosis type 1; CPT = congenital pseudoarthrosis of tibia; MHE = multiple hereditary exostoses.

	N Patients (%)	N LPs (%)	Median (Q1–Q3) Age at Surgery (years)	Median (Q1–Q3) Preoperative LLD (cm)	Median (Q1–Q3) HI (days/cm)	Median (Q1–Q3) TTT (days)	Median (Q1–Q3) Postoperative LLD (cm)	% LPs with One or More Complications	% LPs with One or More Complications (HI > 45 Included)
**Congenital causes**	**41 ** **(82%)**	**59 (84%)**	**14.8**	**7.0**	**50**	**248**	**1.0**	**42%**	**68%**
			**(11.7–16.0)**	**(5.0–9.0)**	**(40–59)**	**(214–300)**	**(0.0–2.5)**		
Idiopathic	8 (16%)	9 (13%)	15.2	4.0	62	302	0.0	22%	100%
			(13.5–16.2)	(4.0–7.0)	(52–67)	(232–318)	(0.0–1.3)		
Proximal hypoplasia *CFD*	12 (24%) *11*	19 (27%) *18*	*15.1* *(10.3–15.4)*	*7.5* *(6.0–9.0)*	*51* *(42–78)*	*223* *(209–251)*	*1.5 * *(0.0–3.0)*	*44%*	*50%*
*DDH*	*1*	*1*	*11.7* *(-)*	*9.0* *(-)*	*63* *(-)*	*316* *(-)*	*1.5* *(-* *)*	*0%*	*100%*
Distal hypoplasia *Fibular hemimelia*	17 (34%) *9*	26 (37%) *18*	*13.4* *(8.6–15.9)*	*8.0* *(6.0–11.0)*	*46* *(39–54)*	*274* *(217–283)*	*1.5 * *(1.0–2.0)*	*56%*	*78%*
*Tibial hemimelia*	*5*	*5*	*14.8* *(14.5–15.9)*	*6.0 * *(5.5–6.0)*	*39* *(37–45)*	*271* *(224–278)*	*0.0 * *(0.0–1.0)*	*60%*	*60%*
*CPMBT*	*3*	*3*	*15.0* *(13.7–15.1)*	*4.5 * *(4.0–5.0)*	*42* *(36–43)*	*211* *(182–239)*	*0.0 * *(0.0–0.0)*	*0%*	*0%*
Skeletal dysplasias	4 (8%)	5 (7%)	16.0 (14.0–16.1)	5.0 (4.5–9.0)	47 (37–55)	256 (192–279)	1.8 (0.5–3.8)	40%	80%
*Ollier’s disease*	*1*	*2*							
*CPT without NF1*	*1*	*1*							
*CPT in NF1*	*1*	*1*							
*MHE*	*1*	*1*							
**Acquired causes**	**9 ** **(18%)**	**11 ** **(16%)**	**15.6**	**6.0**	**56**	**245**	**0.0**	**27%**	**64%**
			**(14.8–16.4)**	**(5.0–9.0)**	**(44–64)**	**(194–299)**	**(0.0–0.0)**		
Infections	3 (6%)	4 (6%)	14.8 (13.4–15.7)	9.5 (6.5–10.0)	67 (50–77)	376 (270–453)	1.8 (0.0–3.0)	25%	75%
Trauma	6 (12%)	7 (10%)	16.0	5.2	52	225	0.0	29%	57%
			(14.8–16.4)	(5.0–6.0)	(30–60)	(186–265)	(0.0–0.0)		
**TOTAL**	**50** **(100%)**	**70** **(100%)**	**14.9** **(12.0–16.0)**	**6.3 ** **(5.0–9.0)**	**51 ** **(40–60)**	**247** **(211–299)**	**0.4** **(0.0–2.0)**	**40%**	**67%**

**Table 2:** Complications classified according to the classification of Lascombes et al. [12]. N = number; EF = external fixator; GA = general anesthesia.

Grade According to Lascombes’ Classification	Type of Complication	N
Grade I	Superficial infection requiring antibiotics	14
	Superficial thrombophlebitis	1
	Temporary nerve palsy	1
Grade IIa	Early union of regenerate	1
	Revision of EF under GA	1
Grade IIb	-	0
Grade IIIa	HI > 45 days/cm	38
	Joint stiffness	9
	Fracture after EF removal	1
	Non-union	1
	Residual angular deformity requiring osteotomy	2
Grade IIIb	Lengthening procedure interrupted and EF removed	2
Grade IVa	-	0
Grade IVb	-	0
	TOTAL	71

**Table 3:** Results of single items of the Short Form 36 (SF-36). PF = physical function; RP = role-physical; BP = bodily pain; GH = general health; VT = vitality; SF = social function; RE = role-emotional; MH = mental health; PCS = Physical Component Summary; MCS = Mental Component Summary.

	PF	RP	BP	GH	VT	SF	RE	MH	PCS	MCS
Mean	82	86	77	69	61	78	65	63	52	44
Median	90	100	80	70	60	75	67	65	54	43
Range	5–100	0–100	35–100	25–95	30–100	25–100	0–100	16–96	29–61	24–58

**Table 4:** Spearman’s rank correlations among SF-36 items and preoperative and postoperative variables. Rho coefficient is reported, (*) indicates coefficient with a *p*-value < 0.10. PF = physical function; RP = role-physical; BP = bodily pain; GH = general health; VT = vitality; SF = social function; RE = role-emotional; MH = mental health; PCS = Physical Component Summary; MCS = Mental Component Summary; LLD = lower limb length discrepancy.

	PF	RP	BP	GH	VT	SF	RE	MH	PCS	MCS
Lengthening cycles	0.14	−0.07	0.04	−0.07	0.09	0.01	−0.04	−0.10	0.06	−0.09
Age at first surgery	−0.05	−0.09	0.06	−0.12	0.05	−0.10	−0.10	−0.10	−0.03	−0.06
Age at questionnaire	−0.21	−0.07	−0.11	−0.37 *	−0.29	−0.17	−0.14	−0.15	−0.21	−0.18
Preoperative LLD	−0.14	−0.09	−0.31 *	−0.26	−0.04	−0.16	−0.10	−0.06	−0.33 *	−0.09
Total lengthening	0.15	−0.07	0.05	−0.08	0.09	0.01	−0.04	−0.10	0.01	−0.09
Complications	0.05	−0.15	0.08	−0.05	−0.01	0.13	−0.06	−0.25	0.08	−0.19
Minor complications	0.02	−0.01	0.14	−0.05	−0.04	0.05	0.04	−0.15	0.08	−0.09
Major complications	−0.04	−0.25	−0.04	−0.10	0.05	0.06	−0.14	−0.27	−0.03	−0.22
Total treatment time	−0.08	−0.21	−0.23	−0.23	−0.01	−0.18	−0.28	−0.19	−0.14	−0.27
Healing index	−0.22	−0.13	−0.25	−0.17	−0.12	−0.21	−0.27	−0.18	−0.23	−0.27
Residual LLD	−0.31 *	−0.20	−0.28	−0.28	−0.15	−0.23	−0.20	−0.17	−0.34 *	−0.19
Follow-up	0.01	0.02	0.02	0.08	−0.05	0.02	0.02	0.01	0.07	−0.01

**Table 5:** Results of single items of the Stanmore Limb Reconstruction Score (SLRS).

	Pain	Sleep	Social	PF	Hygiene	Leisure	Work	Future	Emotions	Cosmetic
Mean	84	73	81	87	96	70	89	73	61	54
Median	87	75	88	95	100	75	100	75	60	50
Range	46–100	13–100	25–100	8–100	25–100	0–100	13–100	25–100	15–100	10–100

## Data Availability

Data are available from the corresponding authors upon reasonable request due to privacy reasons.

## Supplementary Materials

The following supporting information can be downloaded at: https://www.mdpi.com/article/10.3390/children11111322/s1, Table S1: Heterogeneity analysis among the groups of patients observed after the inclusion process. Table S2: Results of Short Form 36 (SF-36) items reporting mean, median, normality test, normative data and comparison. Figure S1: Visual representation of Short Form 36 (SF-36) results comparison with normative data by age and sex. Table S3: Spearman’s correlations among Short Form 36 (SF-36) items and Stanmore Limb Reconstruction Score (SLRS) results. Table S4. Studies that evaluated PROMs after lengthening procedures in children and adolescents.
