# Supplementary material for: Evaluation of Physical and Mental Health in Adults Who Underwent Limb-Lengthening Procedures with Circular External Fixators During Childhood or Adolescence
Source: Children (Basel). 2024 Oct 30;11(11):1322. doi: 10.3390/children11111322 (PMC11592713; doi:10.3390/children11111322)
Supplement: Supplementary file 1 [file children-11-01322-s001.zip › children-3188539-supplementary.pdf]

SUPPLEMENTARY MATERIALS

Table S1. Heterogeneity analysis among the groups of patients observed after the inclusion process. N = number; LLD = lower limb length discrepancy; Q1 = first quartile; Q3 = third quartile; LP = lengthening procedure; HI = healing index; TTT = total treatment time; PROMs = patient reported outcome measures; (*): adjusted residual > ±2.0.

	N	Median (Q1-Q3) LLD (cm)	% patients with > 1 LP	Median (Q1-Q3) HI (days/cm)	Median (Q1-Q3) TTT (days)	% patients with residual LLD > 1.5 cm	% Procedures with one or more complications	% Procedures with one or more complications (HI>45 included)
**Unreachable Patients (excluded)**	67	6.0 (4.0 – 7.5)	4%*	50 (37 – 64)	224 (197 – 273)	10%*	48%	77%
**Patients who filled the PROMs (included)**	50	6.0 (4.0 – 8.0)	24%*	51 (40 – 60)	247 (211 – 299)	32%*	41%	67%
**Patients reachable that did not fill PROMs (excluded)**	61	6.0 (4.0 – 10.0)	16%	54 (41 – 67)	226 (197 – 281)	17%	40%	78%
		p = 0.193	*p = 0.009**	*p = 0.552*	*p = 0.193*	*p = 0.014**	*p = 0.484*	*p = 0.198*

Table S2. Results of Short Form 36 (SF-36) items reporting mean, median, normality test, normative data and comparison. SD = standard deviation; Q1-Q3 = values of first and third quartile; N = number; PF = physical function; RP = role-physical; BP = bodily pain; GH = general health; VT = vitality; SF = social function; RE = role-emotional; MH = mental health; PCS = Physical Component Summary; MCS = Mental Component Summary.

	Shapiro-Wilk p-value	Mean ± SD	Median (Q1-Q3)	Mean ± SD (N) from normative data for age	p-value
**PF**	0.001	82.0 ± 20.4	90 (75 – 95)	95.07 ± 11.01 (724)	0.001*
**RP**	0.001	85.5 ± 24.3	100 (75 – 100)	88.10 ± 27.38 (724)	0.257
**BP**	0.054	77.3 ± 21.2	80 (68 – 100)	80.97 ± 22.33 (727)	0.130
**GH**	0.081	68.7 ± 20.1	70 (60 – 90)	80.54 ± 18.01 (720)	0.001*
**VT**	0.922	60.6 ± 15.0	60 (50 – 70)	59.65 ± 18.32 (725)	0.360
**SF**	0.013	77.5 ± 24.9	75 (50 – 100)	89.18 ± 17.90 (728)	0.001*
**RE**	0.269	64.7 ± 35.9	68 (33 – 100)	87.85 ± 27.04 (724)	0.001*
**MH**	0.612	63.0 ± 17.6	65 (50 – 74)	78.90 ± 14.22 (725)	0.001*
**PCS**	0.001	52.2 ± 7.2	54.0 (49.7 – 57.4)	53.57 ± 7.07 (716)	0.196
**MCS**	0.271	43.9 ± 8.6	43.4 (39.2 – 51.8)	50.67 ± 8.87 (716)	0.001*

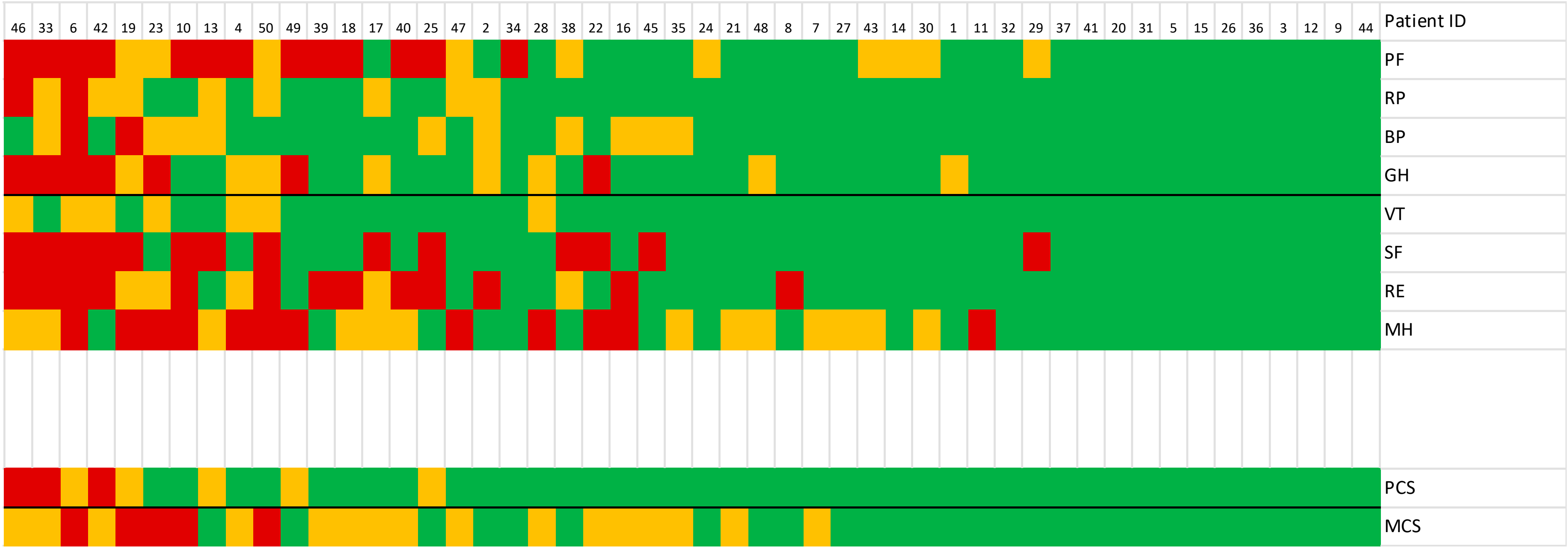

Figure S1. Visual representation of Short Form 36 (SF-36) results comparison with normative data by age and sex. Each column represents a patient, and each square represent a single item of SF-36 and they are ordered from the worst cumulative result (left) to the best cumulative result (right). Green squares indicate scores within ± 1 standard deviation (SD), yellow squares indicate scores within – 1 SD and – 2 SD, red squares indicate scores below – 2 SD. PF = physical function; RP = role-physical; BP = bodily pain; GH = general health; VT = vitality; SF = social function; RE = role-emotional; MH = mental health; PCS = Physical Component Summary; MCS = Mental Component Summary.

Table S3. Spearman's correlations among Short Form 36 (SF-36) items and Stanmore Limb Reconstruction Score (SLRS) results. (*) = rho coefficients with p-value < 0.10; (**) = rho coefficients with p-value < 0.01; (***) = rho coefficients with p-value < 0.001; PF = physical function; RP = role-physical; BP = bodily pain; GH = general health; VT = vitality; SF = social function; RE = role-emotional; MH = mental health; PCS = Physical Component Summary; MCS = Mental Component Summary.

	Pain	*Sleep*	*Social*	*PF*	*Hygiene*	*Leisure*	*Work*	*Future*	*Emotions*	*Cosmetic*	
**PF**	**0.42****	0.13	**0.62*****	**0.80*****	**0.57*****	0.29*	**0.51*****	**0.57*****	**0.59*****	**0.46*****	***PF*** $R^2_{adj} = 0.80$ p = 0.001
**RP**	0.27*	0.16	**0.47*****	**0.50*****	0.32*	**0.38****	**0.56*****	**0.45*****	**0.62*****	0.18	***Work, Emotions*** $R^2_{adj} = 0.55$ p = 0.001
**BP**	**0.87*****	0.33*	**0.48*****	**0.48*****	**0.51*****	**0.37****	**0.58*****	**0.48*****	**0.56*****	0.19	***Pain, Social, PF(–), Work*** $R^2_{adj} = 0.80$ p = 0.001
**GH**	0.24*	**0.42****	0.34*	0.35*	**0.38****	**0.38****	**0.47*****	0.31*	**0.58*****	0.25*	***Work, Emotions*** $R^2_{adj} = 0.41$ p = 0.001
**VT**	0.26*	**0.38****	0.34*	**0.43****	0.35*	**0.45****	0.33*	0.35*	**0.60*****	**0.54*****	***Emotions, Cosmetic*** $R^2_{adj} = 0.44$ p = 0.001
**SF**	**0.37****	0.21	**0.90*****	**0.59*****	0.34*	**0.41****	**0.50*****	**0.50*****	**0.55*****	0.24	***Social*** $R^2_{adj} = 0.80$ p = 0.001
**RE**	**0.49*****	**0.36****	**0.60*****	**0.61*****	**0.52*****	**0.37****	**0.80*****	**0.55*****	**0.77*****	**0.42****	***Work, Emotions*** $R^2_{adj} = 0.77$ p = 0.001
**MH**	**0.37****	0.35*	0.33*	**0.42****	**0.37****	0.22	0.17	**0.44****	**0.64*****	0.24*	***Emotions*** $R^2_{adj} = 0.47$ p = 0.001
**PCS**	**0.55*****	0.25*	**0.51*****	**0.55*****	**0.45****	**0.46*****	**0.53*****	**0.46*****	**0.54*****	0.23	***PF, Work*** $R^2_{adj} = 0.63$ p = 0.001
**MCS**	**0.46*****	**0.46*****	**0.62*****	**0.56*****	**0.42****	**0.41****	**0.54*****	**0.54*****	**0.83*****	**0.38****	***Social, Emotions*** $R^2_{adj} = 0.73$ p = 0.001
	**BP** $R^2_{adj} = 0.76$ p = 0.001	**MCS** $R^2_{adj} = 0.16$ p = 0.003	**RP(–) SF RE** $R^2_{adj} = 0.83$ p = 0.001	**PF** $R^2_{adj} = 0.80$ p = 0.001	**PF** $R^2_{adj} = 0.53$ p = 0.001	**VT** $R^2_{adj} = 0.16$ p = 0.002	**BP(–) RE PCS** $R^2_{adj} = 0.74$ p = 0.001	**RP BP** $R^2_{adj} = 0.30$ p = 0.001	**RE MH** $R^2_{adj} = 0.72$ p = 0.001	**VT** $R^2_{adj} = 0.24$ p = 0.001	

Table S4. Studies that evaluated PROMs after lengthening procedures in children and adolescents. N = number of patients; LP = lengthening procedure; PROMs = Patient-Reported Outcome Measures; QoL = quality of life; HRP = healthy reference population; N/A = not available; LLD = lower limb length discrepancy; CDI = Children's Depression Inventory by Kovacs 1985; CHISRS = Children Health Information Service Rand Scale by Eisen et al. 1979; PHBQ = Post Hospitalization Behavior Questionnaire by Vernon et al. 1966; OSIQ = Offer Self-Image Scale Questionnaire by Offer et al. 1992; PedsQL = Pediatric Quality of Life Inventory 4.0 Generic Core Scales; TAAQOL = TNO-AZL Questionnaire for Adult's Health-related Quality of Life by Theunissen et al. 1998; ; RCMAS = Revised Children Manifest Anxiety Scale by Reinolds and Richmond 1976; SPIQ = Speedy Performance of IQ; YSR/CBCL = Youth Self-report and Child Behavior Checklist by Achenbach 1991; STAI (X) = State-Trait Anxiety Inventory by Spielberger 1983; WHOQoL-BREF = Quality of Life Test-BREF developed by World Health Organization in 1995; DSA = Depression Scale for adolescents by Zung et al. 1965.

**Author (year)**	**N**	**Etiology**	**Age at LP (mean, range)**	**Years since EF removal (mean, range)**	**Psychological support during LP**	**PROMs**	**Results**
Ghoneem et al. (1996)<br>Canada	45	30 congenital<br>15 acquired	12.0<br>(3.0 – 18.0)	3.0	Yes	CDI<br>CHISRS<br>PHBQ	Physical function subscale functional score for all the patients was 0.7 point (range, 0–5).<br>42 children (93%) had no limitations in daily activities, 3 children (7%) had some limitation in activities.<br>Overall good normal psychological functioning.<br>83% of children treated between 6-12 years of age were willing to be treated again with Ilizarov method, compared with 45% of children treated between 13-18 years of age.
Martin et al. (2003)<br>England	15	7 congenital<br>8 acquired	(11.0 – 18.0)	0.1	Yes	CDI<br>OSIQ	The prevalence of patients with high CDI scores, indicating possible depression, decreased significantly from 33% before surgery to 7% one month after EF removal.<br>The prevalence of patients with low body image (OSIQ below – 1 SD) slightly increased from 40% preoperatively to 53% one month after frame removal.
Montpetit et al. (2009)<br>Canada	52	43 congenital<br>9 acquired	13.0<br>(5.0 – 21.0)	0.3	No	PedsQL	Scores decreased in all items until mid-distraction phase and then an improvement was reported at 3 months after frame removal, in particular in the emotional functioning.
Moraal et al. (2009)<br>Netherlands	37	22 congenital<br>15 acquired	13.2<br>(8.0 – 20.0)	6.9<br>(2.0 – 14.1)	No	TAAQOL	All patients had lower scores in gross motor, pain and vitality compared to HRP.<br>Patients with residual LLD > 2 cm had lower scores in gross motor, sleep and depressive feelings compared to other patients.<br>No influence on scores by etiology and number of LPs.
Niemelä et al. (2008)<br>Sweden	30	23 congenital<br>7 acquired	11.3<br>(6.0 – 13.0)	1.0	Yes	RCMAS<br>I think I am<br>CDI<br>SPIQ<br>YSR/CBCL<br>STAI (X)	Higher prevalence of extremely satisfied children among older patients; children in the 10-12 age range showed the highest levels of anxiety and depression (although not significantly different).<br>Self-esteem improved after treatment, becoming comparable with controls.<br>No higher prevalence of depression than in the control group before the procedure; depression worsened postoperatively only in one patient (3%).<br>Behavior improved in all patients after procedure.<br>High state anxiety before procedure in parents.
Pawik et al. (2021)<br>Poland	58	N/A	N/A	3.4<br>(over 2.0)	No	WHOQoL-BREF	Patients with residual LLD > 1 cm had higher prevalence of low scores in overall QoL, self-assessment of health, physical and mental functioning compared to patients with residual LLD < 1 cm.<br>All treated subjects assess their QoL and their health less favorably than those in the control group.<br>Etiology of conditions included not clearly specified (defined as isolated shortness of the tibia in all cases).
Ramaker et al. (2000)<br>Netherlands	26	17 congenital<br>8 acquired<br>1 unknown	(6.0 – 17.0)	3.0<br>(1.3 – 5.6)	Yes	Zung DSA<br>STAI (X)	No significant changes in depression or anxiety before and after the procedure.<br>87% would choose the LP again.<br>Almost a quarter of the patients still had complaints about their leg after EF removal.
